# Human Polyomavirus Reactivation: Disease Pathogenesis and Treatment Approaches

**DOI:** 10.1155/2013/373579

**Published:** 2013-05-02

**Authors:** Cillian F. De Gascun, Michael J. Carr

**Affiliations:** ^1^Department of Virology, Frimley Park Hospital, Frimley, Surrey GU16 7UJ, UK; ^2^National Virus Reference Laboratory, University College Dublin, Belfield, Dublin 4, Ireland

## Abstract

JC and BK polyomaviruses were discovered over 40 years ago and have become increasingly prevalent causes of morbidity and mortality in a variety of distinct, immunocompromised patient cohorts. The recent discoveries of eight new members of the *Polyomaviridae* family that are capable of infecting humans suggest that there are more to be discovered and raise the possibility that they may play a more significant role in human disease than previously understood. In spite of this, there remains a dearth of specific therapeutic options for human polyomavirus infections and an incomplete understanding of the relationship between the virus and the host immune system. This review summarises the human polyomaviruses with particular emphasis on pathogenesis in those directly implicated in disease aetiology and the therapeutic options available for treatment in the immunocompromised host.

## 1. Introduction

Polyomaviruses (PyV) are small (diameter 40–50 nm), nonenveloped, circular, double-stranded DNA viruses of the family *Polyomaviridae*. To date, 32 PyV species have been described, ten of which have been reported to infect humans (HPyVs), although not all of those have yet been definitively linked with disease [1]. In recent years, there has been a significant increase in the study of HPyVs as eight novel species have been discovered since 2007: KIPyV [2], WUPyV [3], Merkel cell polyomavirus (MCV) [4], HPyV6 [5], HPyV7 [5], trichodysplasia spinulosa-associated PyV (TSV) [6], HPyV9 [7], and MW PyV/HPyV10 [8, 9]. Prior to this, most clinicians would have been familiar with JC PyV (JCV) and BK PyV (BKV), the first two HPyVs, which were discovered in 1971 in patients who were immunosuppressed: JCV was identified in brain tissue from a patient with progressive multifocal leukoencephalopathy (PML) [10] and BKV from the urine of a renal transplant patient [11]. Seroprevalence studies subsequently demonstrated that both JCV and BKV were far more prevalent in the general population than the incidence of the diseases that they caused (PML and BKV-associated nephropathy (BKVN), resp.) [12]. The increased incidence of JCV/PML in association with the HIV-1 pandemic and the emergence of BKV/BKVN in association with renal transplantation (and haemorrhagic cystitis in bone marrow transplant recipients) highlighted the importance of the host immune system in the control of these latent infections and the pathogenesis of these diseases [13, 14].

Until the early part of this century, the JCV/BKV pattern of disease has been the hallmark of HPyV infection: asymptomatic primary infection occurring almost universally in childhood, from which time, the virus remains latent in the human host; viral reactivation—as evidenced by the presence of viral DNA in urine or, less frequently, blood—occurring intermittently throughout life but rarely causing disease in the otherwise immunocompetent host; and occasional cases of PyV-associated disease in the profoundly immunosuppressed, susceptible host. Recent events and discoveries, however, suggest it may be time to reconsider this paradigm. There have been multiple reports of the development of PML as a side effect of the immunomodulatory therapies (monoclonal antibodies) natalizumab [15–17], rituximab [15, 18], efalizumab [15, 19], and infliximab [20] used to treat various chronic medical conditions. In addition, eight novel HPyVs have been discovered, at least one of which (MCV) does not appear to follow the traditional disease course described above for JCV and BKV [4]. Taken in conjunction, these findings provide an opportunity to reevaluate HPyVs and their role in human disease. This review will focus primarily on JCV, BKV, MCV, and TSV on account of their now well established disease associations. However, it will also discuss the clinical and epidemiological data that currently exist for HPyVs 3, 4, 6, 7, 9, and 10.

## 2. Classification

**Figure 1:**
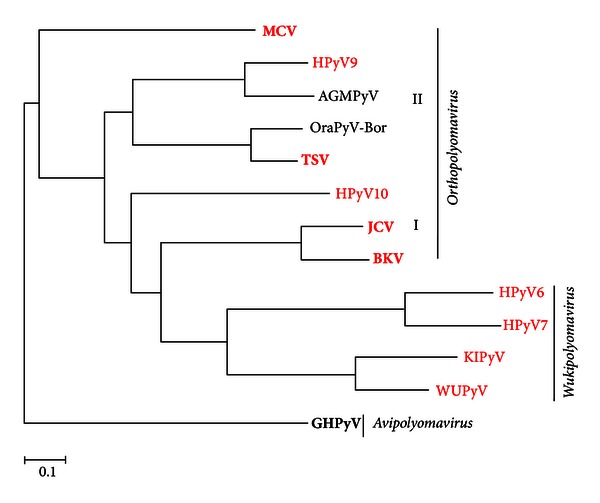
Phylogenetic relationships of the human polyomaviruses. Human polyomaviruses are presented in red with those associated with clinical disease in bold. The mammalian genera within the *Polyomaviridae* family: *Orthopolyomavirus* and *Wukipolyomavirus* and the single *Avipolyomavirus *genus member (employed as an outgroup) are indicated. Maximum likelihood phylogenetic analysis was performed on polyomaviral whole genome nucleotide sequences. Abbreviations and GenBank accession numbers employed as follows: AGMPyV, African green monkey polyomavirus (NC004763); BKV, BK polyomavirus (NC001538); goose haemorrhagic polyomavirus (GHPyV) genus (NC004800); HPyV6, human polyomavirus 6 (NC 004800); HPyV7, human polyomavirus 7 (HM011565); JCV, JC polyomavirus (NC001699); KIPyV, KI polyomavirus (NC 009238); MCV, The Merkel cell polyomavirus (HM011539); HPyV9, human polyomavirus 9 (HQ696595); MWHPyV, The Malawi polyomavirus (JX262162); TSV, trichodysplasia spinulosa-associated polyomavirus (GU989205); WUPyV, WU polyomavirus (NC009539).

The family *Polyomaviridae* came into existence in 2000, when the International Committee on Taxonomy of Viruses formally split the genera of the *Papovaviridae* family—the polyomaviruses and papillomaviruses—to form two new families, *Polyomaviridae* and *Papillomaviridae* [21]. The name polyomavirus, meaning “many tumours” is derived from Greek, and based on the fact that the first polyomavirus isolated—murine polyomavirus—caused the formation of multiple tumour sites when inoculated into newborn mice [22]. Indeed, injection of BKV and JCV into rodents also leads to the formation of multiple tumours [13, 23]. However, until the discovery of MCV, there was no direct association between the HPyVs and tumour formation in humans. The ten known HPyVs, adult seroprevalence, clinical disease and risk groups are summarized in Table 1. The family *Polyomaviridae* now comprises two mammalian genera, *Orthopolyomavirus* (consisting of two separate lineages: I and II) and *Wukipolyomavirus,* an avian genus, *Avipolyomavirus*, and a fifth distinct group—yet to be named—of which HPyV10 is currently the only member [13], see Figure 1. Of note, the HPyVs do not form a distinct cluster: JCV and BKV are found in *Orthopolyomavirus* lineage I, with MCV, TSV, and HPyV9 in lineage II. The remaining human PyVs (excluding HPyV10) are in the *Wukipolyomavirus* genus [1]. 

**Figure 2:**
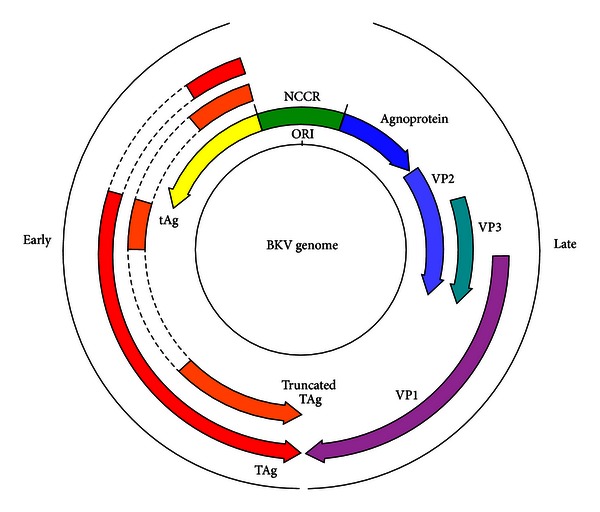
Schematic diagram illustrating the organisation of the dsDNA genome of BK virus. The open reading frames are represented by arrows with alternative splicing events highlighted by dashed lines. The origin of replication (ORI) within the noncoding control region (NCCR), from which transcription of early and late mRNAs proceeds, is indicated. The agnoprotein and truncated T antigen genes have not been described in all polyomaviruses.

The outer shell of the PyV capsid is constructed of 360 molecules of the major capsid protein VP1, organised into 72 pentamers, with each pentamer associated with a single copy of the minor structural protein VP2 or VP3. Only VP1 is exposed on the surface of the capsid and thus determines receptor specificity [23]. VP2 and VP3 are believed to play a role in stabilising the virus particle outside of the host cell, and—following alterations in the capsid structure that take place on cell entry—in traversing the intracellular interior [13, 23]. The genomic structure is highly related among the primate PyVs with a genome of around 5000 base pairs in length encoding six major viral proteins divided into three regions: the early coding region, the late coding region; and the noncoding control region (NCCR; see Figure 2). Each half of the PyV genome carries approximately half of the open reading frames, with replication proceeding in a bidirectional, temporally defined manner from the origin of replication (ORI) within the NCCR so that early and late transcribing regions are physically separated by the NCCR [23]. The PyV early proteins are translated from a series of alternative splicing events derived from a common mRNA precursor. The early coding region—transcribed before DNA replication begins—encodes large T antigen (TAg) and small t antigen (tAg). The BKV genome encodes three early proteins including the truncated tumour antigen (truncTAg) expressed from an alternatively spliced BKV early mRNA [24]. The late coding region—expressed after the onset of DNA replication—encodes the three viral structural proteins, VP1, VP2, and VP3, as well as the accessory agnoprotein. The PyV tumour antigens are multifunctional regulatory proteins that are essential for viral replication: in addition to driving the host cell towards the S phase of the cell cycle so that viral replication can occur; they also initiate viral DNA replication and they regulate transcription from the host and viral genomes. The agnoprotein appears to be multifunctional, with highly varied roles attributed to it, from viral transcription regulation to inhibition of host DNA repair to functioning as a viroporin [25–28].

## 3. JC Polyomavirus

### 3.1. Modes of Transmission and Epidemiology of JCV

The first case of demyelinating disease described with the term PML was found in a patient with chronic lymphocytic leukaemia and Hodgkin's lymphoma in 1958 [29], but accounts of potential cases can be traced as far back as 1930 [23, 30, 31]. A viral aetiology for PML was first proposed in 1959, based on observations of inclusion bodies in the nuclei of damaged oligodendrocytes [32]. However, it was not until 1971 that the causative agent was identified [10]. Padgett and colleagues isolated the virus from a mixed culture of glial cells and named it after the initials of the patient. The capacity of JCV to cause haemagglutination of human type O erythrocytes [33] facilitated seroprevalence studies, which demonstrated a worldwide distribution [34] and revealed that a large percentage of the population were asymptomatically infected before adulthood [35, 36]. Subsequently, more recent studies have confirmed these findings, with a reported prevalence for JCV of ~50%–80% in the general population [12, 37–39], although these rates vary among populations and age groups [40]. In addition, it has been shown that at any given time, approximately one-fifth of the population sheds JCV in urine [14]. Virus has also been detected in stool samples and is prevalent in sewage and rivers worldwide [41–45] raising the possibility of transmission through ingestion of nonsterile water. Full-length genome sequencing has identified seven JCV types, numbered 1–8 (type 5 was found to be a minor member of type 3), each with multiple subtypes [46]. The different types of JCV are associated with distinct human populations [46] and have been used to map population movements [47–50]. It has been hypothesised that type 6 is the original JCV type and that JCV coevolved with human populations, diverging as humans migrated out of Africa [51]. Types 1 and 4 are generally associated with Europeans, types 3 and 6 with Africans, type 2A with Asians, and 2D and 7C with Asians and South Asians. Types 2E, 8A, and 8B are found in Western Pacific populations with type 8A found only in Papua New Guinea [52–54]. Subtype 2B, found more often in Asians and Eurasians, has been associated with an increased risk of PML [46, 55]; type 4 has been associated with a lower disease risk [56].

In the majority of individuals, JCV infection is controlled by the healthy immune system [23], an interpretation supported by the epidemiology of PML. PML is an AIDS-defining illness, occurring in 3%–5% of HIV-infected individuals [14]. However, the rarity of PML prior to the AIDS pandemic—when it was associated primarily with B cell lymphoproliferative disorders [57, 58]—indicates that a reduction of CD4+ T-cells leads to a lack of immune control of JCV. In addition, non-HIV-related CD4+ T-cell reduction has also been associated with PML [59, 60]. Conversely, a cytotoxic T-cell response has been associated with greater control of JCV and longer PML survival rates [61, 62]. Furthermore, the use of highly active antiretroviral therapy (HAART) for the treatment of HIV has led to a reduced rate of PML in HIV-infected individuals despite having no demonstrable direct effect on JCV replication [23].

Indeed, both the EuroSIDA [63] and Swiss HIV cohort [64] studies have reported the clinical benefit of HAART on the incidence of PML, with reduced annual post-HAART rates of 0.6-0.7 per thousand. In immunocompromised individuals who are not infected with HIV, PML remains rare. In immunocompromised individuals who are not infected with HIV, PML remains rare. In a large population-based investigation, Amend and colleagues reported annual rates per 100 000 of 11.1 in chronic lymphocytic leukaemia, 10.8 in autoimmune vasculitis, 8.3 in non-Hodgkin's lymphoma, and 2.4 in systemic lupus erythematosus [63]. Studies in patients with rheumatoid arthritis have reported rates of 0.4 [66] to 1.0 [67] per 100,000, with the latter Swedish study also reporting a rate in the general population of 0.3. In individuals with multiple sclerosis (MS), however, the risk of PML has increased with the use of monoclonal antibodies, in particular natalizumab: the incidence of PML has risen from 0.09 per 1000 (for those who are anti-JCV negative) to 11.1 per 1000 for those who have received 24–48 months of natalizumab [68]. Similarly, in bone marrow transplant patients, the risk of PML appears to have surpassed that of HIV infected individuals, with both Amend et al. (35.4 per 100,000) [65] and Mateen and colleagues (1.24 per 1000) [69] reporting PML incidence rates in this group that are greater than that recorded in the EuroSIDA and Swiss HIV cohorts.

In spite of the fact that JC virus was identified as the aetiological agent of PML over 40 years ago, the definitive route of viral transmission and subsequent transport to the brain remain to be fully elucidated. The capacity of the virus to interact with B cells in the brain and replicate at low levels within B cells suggested a probable haematogenous route of CNS transmission [70–73]. Additional evidence that tonsillar stromal cells could be one of the initial sites of infection [57] led to the first working hypothesis that following primary infection—either via respiratory or oral acquisition—the virus is trafficked by infected lymphocytes from stromal or immune cells in the upper respiratory system to the bone marrow or kidneys, where it can persist for the life of the host. CD34+ haematopoietic stem cells harbor the virus in the bone marrow, and these cells migrate into the peripheral circulation and undergo differentiation to pre-B and mature B cells, augmenting JCV expansion [74]. Following immunosuppression, the virus mobilises from the bone marrow, and crosses the blood-brain barrier (BBB), with lytic infection commencing when the oligodendrocytes become infected [58]. 

The second working hypothesis for the pathogenesis of PML proposes that either the brain or the kidney may serve as a site of latency, indicating that JCV is already present in the brain at the time of the immune insult and that PML results from a loss of immune surveillance. In this model, JCV reaches the brain—possibly through B cells—during the viral dissemination that occurs following primary infection, reaching glial cells where it remains latent [23]. In support of this hypothesis, JCV DNA has been found in the brains of both healthy and immunocompromised patients without PML and other neurological disorders [75–78]. However, this pathway does not account for the very low incidence of PML in allograft recipients who are immunosuppressed for substantial periods of time for graft protection [23].

### 3.2. Pathogenesis of PML

Regardless of the site of viral latency or which of the above models is correct, the fundamental premise is that at least four events must occur before latent JCV can cause lytic infection of oligodendrocytes in the brain: (i) the host immune system must be compromised; (ii) the viral NCCR (discussed in more detail below) must acquire changes that increase viral transcription and replication in both B cells and glial cells; (iii) transcription factors that bind to the recombined NCCR sequence motifs must be present and/or upregulated in infected haematopoietic progenitor, B cells, and/or glial cells; (iv) free virus or virus in B cells must cross the BBB and be carried to the brain, where the virus is passed to oligodendrocytes and lytic infection takes place [23]. These events may occur in the bone marrow, in CD34+ lymphocyte precursors or B cells in the periphery, or in the brain. Significantly, in cases of PML, latent JCV DNA has been demonstrated in pathologic tissue from lymph, spleen, or bone marrow biopsies taken months to years before the patient developed neurological disease [79].

The PyV NCCR is the most variable portion of the viral genome, both within a single virus, as well as across genera of viruses [80–86]. It is thought to be the main determinant of cell type specificity, containing the origin of replication and numerous transcription factor binding sites [23]. In JCV infection, the NCCR varies greatly between isolates from PML patients. However, an “archetype” sequence (also known as CY) has been isolated from urine specimens from both PML patients and healthy individuals but is rarely found in PML lesions [23]. The NCCR from the original Mad-1 isolate of JCV contains an enhancer element that exists as a 98-bp direct tandem repeat and therefore contains duplicate TATA boxes, which can position mRNA start sites [87, 88] as well as multiple transcription factor binding sites [89]. The Mad-1 NCCR tandem repeat structure has been termed the “prototype” JCV NCCR sequence and is composed of three blocks of sequence, named “a,” “c,” and “e” with the TATA box found in “a.” Although Mad-1 was the first isolated NCCR sequence, many JCV isolates from PML patients do not possess the second TATA box, indicating it may not be essential for viral replication [90, 91]. The NCCR sequence of the “archetype” JCV is composed of a single copy of the 98-bp repeat of a-c-e, with 23-bp (“b”) and 66-bp (“d”) sequence blocks between “a,” “c,” and “e” to yield an a-b-c-d-e structure. However, archetype virus is rarely associated with PML [92]. Thus, the consistent isolation of tandem repeat-like NCCR sequences including the 98-bp tandem repeat in PML lesions strongly suggests this structure plays an important role in disease pathogenesis [82, 91, 93–95]. As a general rule, prototype and prototype-like sequences are generally found in PML tissue, while kidney-and urine-derived NCCR sequences are normally identical to archetype [23]. It has been proposed that all JCV isolates contain NCCRs that derive from the archetype sequence [92, 96, 97]; however, a mechanism for this derivation in the host has yet to be determined. Nonetheless, the prevailing disease pathogenesis model holds that the archetype-like sequences are transmitted from person-to-person and then undergo deletions and duplications within the infected host, leading to PML-like NCCR sequences which traffic to the brain. This “rearrangement” of the NCCR may take place in lymphoid cells, like B cells, since they possess the required enzymes for immunoglobulin gene rearrangement. Indeed, prototype-like sequences have been detected in lymphocytes from peripheral blood [57, 73, 98, 99] and the bone marrow [58, 94, 100]. Regardless of how the repeat NCCR variants are generated, this form of JCV is the pathogenic form that has repeatedly been isolated from PML. Compared with the archetype, this sequence contains significantly more transcription factor binding sites, which are essential to viral gene expression. Specifically, the archetype sequence does not contain Spi-B-binding sites, which are important for early viral gene expression [101], and possesses a reduced number of NF-1 binding sites, which are essential for fully activating viral transcription in the brain and cells of the lymphoid system. Spi-B is a transcription factor that binds to sequences in the JCV promoter/enhancer [74] and has been shown to be upregulated in B cells, glial cells, and haematopoietic progenitor cells in which JCV can replicate. The expression of Spi-B is also upregulated in patients with multiple sclerosis who are treated with the monoclonal antibody natalizumab (discussed below) [74]. NF-1 is a nuclear transcription factor and a cell-specific regulator of JCV promoter/enhancer activity. In humans, the NF-1 family of DNA-binding proteins is encoded by four discrete genes, one of which is NF-1 class X (NF-1X). NF-1X has also been shown to be upregulated in B cells, glial cells, and haematopoietic progenitor cells in which JCV can replicate [101–103]. These data suggest that changes in transcription factors can affect viral transcription during the maturation process of B cells. 

Over the past decade, several immunomodulatory therapies, used for the treatment of autoimmune conditions, have been associated with cases of PML [15–20]. The known mechanism of action of each of these therapies has shed light on the host immune control of JCV. Natalizumab is a humanised monoclonal antibody for the treatment of relapsing-remitting multiple sclerosis (RRMS). The antibody binds the *α*4 chain of the *α*4/*β*1 and *β*7 integrin dimer also known as very late antigen-4 (VLA-4) [104]. VLA-4 mediates cell migration and infiltration in immune signaling, through binding its ligand—the vascular cell adhesion molecule (VCAM)—and facilitating the extravasation of leucocytes through endothelial cells to the sites of inflammation. In RRMS, the aim of the monoclonal antibody is to prevent leucocyte infiltration into the brain. However, natalizumab treatment also prevents developing B cells from attaching to a VCAM, forcing them to migrate from the bone marrow [74] and resulting in an increase in CD34+ progenitor cells in both the bone marrow and peripheral blood [105] and of factors involved in B cell differentiation, including Spi-B, in the peripheral blood [106]. Spi-B is also increased in CD34+ cells and B cells in natalizumab-treated patients. The risk of PML increases as treatment progresses, and the incidence of PML is estimated to be approximately 3.85 per 1000 patients treated with more than 24 infusions. Rituximab is an anti-CD20 humanised monoclonal antibody that fixes complement. Binding of CD20, an antigen expressed on B cells, results in downregulation of the B cell receptor and cytolytic apoptosis of CD20+ cells [107], resulting in depletion of CD20+ cells in the peripheral blood and cerebrospinal fluid (CSF) [14, 108]. In this setting, pre-B and B cells may be mobilised from the bone marrow and lymph nodes to replace CD20+ cells, leading to higher levels of CD34+ progenitors in the peripheral blood [14]. Efalizumab is a humanised monoclonal antibody against CD11a, a subunit of the leucocyte function-associated antigen type 1 (LFA-1), a T-lymphocyte adhesion molecule. LFA-1 binds intercellular adhesion molecule 1 (ICAM-1) which allows migration of T lymphocytes from circulation into sites of inflammation [109]. Efalizumab also downmodulates expression of VLA-4 resulting in T-cell hyporesponsiveness [110]. The drug was withdrawn from the market due to the occurrence of PML at an incidence of approximately 1 in 500. Infliximab is a humanized monoclonal antibody against tumour necrosis factor alpha (TNF-*α*) [111] that also induces apoptosis in TNF-*α* producing T-cells [112, 113]. The drug has been associated with an increase in infections or reactivation of latent infections [114], probably due to a blockage of TNF-*α* and T-cell reduction.

Finally, it should be noted that the rate of JCV disease in HIV-infected individuals remains significantly greater than in patients with other underlying causes of immunosuppression [23]. This is believed to be due to several factors: the duration and extent of immunosuppression, changes in cytokine secretion induced by HIV, viral interactions in coinfected cells and increased BBB permeability allowing for B cells infected by JCV to enter the brain [115]. Briefly, in HIV infection, the CD8+ T-cell response required to control JCV infection [116–120] is suboptimal because of the depletion in the CD4+ T-cells required to maintain that response [121]. In addition, HIV Tat protein has been shown to increase transcription from JCV [41, 122–128]; indeed, archetype JCV can replicate in cells expressing HIV Tat [29, 125, 129]. Furthermore, HIV infection of the brain causes upregulation of cytokines that attract lymphocytes [130] as well as an increase in cell adhesion molecules that may facilitate BBB crossing of JCV-infected cells. Finally, the astrocyte and neuronal damage caused by HIV proteins [131–135] lead to increased inflammation and further infiltration by JCV-infected lymphocytes, which may facilitate the onset of PML [23].

### 3.3. JCV-Associated Clinical Disease

The classic triad of PML consists of cognitive impairment, visual deficit and motor dysfunction [74], although symptoms and clinical presentation may vary based on the location and size of the lesion(s). Patients typically present with motor deficits, altered level of consciousness, ataxia, and visual symptoms [136, 137]. Seizures have been reported in PML, but this is believed to be due to the location of the lesions and does not herald a poorer prognosis [137]. Atypical (defined as non-PML) CNS presentations of JC infection have been described. JCV encephalopathy, indicating JC virus infection of the gray matter of the brain, has been reported in an HIV-negative woman with a history of lung cancer [138]; the extension of classic PML lesions into gray matter has also been described [139, 140]. JCV has also been implicated as a causative agent of meningitis in both immunocompromised and immunocompetent individuals [138]. Although not typically part of the routine screen for “viral meningitis” patients, one study has reported a prevalence of 1.5% for JCV in a mixed (immunocompetent and immunocompromised) cohort [141].

JCV-granule cell neuronopathy (JCV-GCN): while changes—enlarged and hyperchromatic nuclei [142]—in the granule cell layer of the cerebellum have been long recognized in PML it was unclear whether these cells were infected by JCV or the victims of collateral damage from the destruction of glial cells. However, in 2003, productive infection of granule cell neurons in the cerebellum was finally described, albeit in the presence of classic PML [143]. Subsequently, JCV was found in the brain of a patient with cerebellar atrophy in the absence of white matter PML lesions. JCV-GCN was proposed to be a novel syndrome distinct from PML and has since been reported in both HIV-positive and HIV-negative patients. Interestingly, the comparison of CSF-isolated virus and cerebellar virus NCCRs from a patient with AIDS showed differences in transcription factor binding-sites [144].

Magnetic resonance imaging (MRI) is the imaging modality of choice if a clinical diagnosis of PML is suspected, with lesions typically manifesting as high-signal intensity on T2-weighted and FLAIR sequences [23]. The lesions are usually multifocal, bilateral, and asymmetrical, involving the uncinate fibres, sparing the gray matter, and demonstrating a predilection for the posterior parts of the brain, although they may occur anywhere [145, 146]. The lesions may appear hypointense on T1-weighted images and do not enhance with the administration of gadolinium, as there is very little or no inflammation [147]. In the early stages of disease, the lesions are often subcortical, subsequently spreading to deep periventricular white matter [147]. Radiological findings alone are not sufficient to confirm a diagnosis of PML. Antibody testing is not currently of diagnostic significance after the onset of symptoms, although it may be used in risk stratification protocols for patients commencing immunomodulatory therapy [23]. The confirmatory test for suspected PML is the demonstration of JCV DNA in the CSF or brain by PCR. Detection of JCV DNA in blood is not of diagnostic significance as viraemia may be present in the absence of PML, and a percentage of PML patients are not viraemic [148]. The specificity of quantitative PCR can be optimised by targeting unique sequences within the JCV T antigen gene that are necessary for infection. In addition, the detection sensitivity of some assays can be as low as 10 copies/mL [149]. The prognostic significance of the magnitude of the viral load in the CSF has not been established [150]. Of note, other changes in the CSF in PML tend to be nonspecific, with a mild increase in protein, but a normal cell count and normal glucose. Interestingly, in the era of HAART and in those patients with MS in whom the immune system is relatively intact, the copy numbers of JCV can be quite low and difficult to detect [151, 152]. In this situation, brain biopsy may be indicated, as the MRI appearance is not pathognomonic for the disease. In brain tissue, JCV infection can be demonstrated by immunohistochemistry, *in situ* hybridization, or PCR analysis [153].

### 3.4. Association of JCV with Human Cancer

JC virus has the capacity to transform cells in culture and induce tumours of neural origin in animals, including rodents and non-human primates [154–156]. In human cancer, however, the data are less conclusive and conflicting reports of the presence of the JCV genome and the T antigen in tumours of both neural and nonneural origin exist. A comprehensive review of the available data in this controversial area is beyond the scope of this report. However, Del Valle and colleagues have recently performed such a review [157]. Although the authors ultimately conclude that JCV involvement in the genesis of neural tumours is a possibility that can neither be confirmed nor excluded at this time, they do highlight the intriguing fact that the cellular signaling pathways that have been identified as targets of JCV TAg in molecular experiments and in experiments with JCV early region transgenic mice are the same pathways that are observed to be dysregulated in human tumours that are immunopositive for TAg [157]. Ultimately, given the prevalence of JCV in the general population, large-scale epidemiological studies will be required to fully investigate the role—if any—of JCV in human cancers [23].

## 4. BK Polyomavirus 

### 4.1. Modes of Transmission and Epidemiology of BKV

BK virus (BKV) was first isolated from a Sudanese renal transplant recipient (initials BK) with ureteral stenosis [11]. BKV acquisition is thought to occur subclinically early in childhood via the respiratory route, or accompanied by mild illness, such as tonsillitis, following contact with aerosols or fomites [158]. Seroconversion to BKV has been demonstrated in paired sera from children hospitalised with acute upper respiratory tract infection with multiple nonintegrated BKV genomes also detected in tonsillar tissue [159]. Evidence also exists to support other transmission modes for BKV, particularly, the faeco-urino-oral route and BKV seroconversion following organ transplantation, particularly in renal allograft recipients, has been established [44, 160–162]. BKV acquisition via semen, blood transfusion, and transplacental vertical transmission has also been put forward, with conflicting results in the latter case [163–168]. Population-based BKV seroprevalence studies indicate that 80%–90% of children are exposed and infected by ten years of age with a median age of 4-5 years [12, 39]. Waning of BKV immunity following the establishment of an infection has been suggested by decreases in antibody titres throughout life [12, 37]. This contrasts with serological correlates of JCV immunity, which remain stable and increase during life suggesting that differing transmission routes for each PyV and/or heterotypic immune responses to prior BKV exposure may afford some protection to infection from subsequent immunologic challenge with JCV [169]. 

There are four distinct serotypes and subtypes (genotypes) of BKV: I, II, III and IV with subtype I (the most prevalent) distributed worldwide, subtype IV in East Asia and Europe, and subtypes II and III rarely described [170]. BKV subtypes are routinely distinguished based on viral capsid protein VP1 nonsynonymous nucleotide polymorphisms and putative antigenic determinants of the BKV subtypes have been mapped within N-terminal residues 61–83 [171, 172]. Geographical separation of subgroups within BKV subtypes has been described with genetic studies showing subgroup Ia is most prevalent in Africans and the presumed ancestral subtype that coevolved with humans in an out of Africa dispersal, subgroup Ib1 significantly higher in Southeast Asians, Ib2 in Europeans and West Asians and Ic in Northeast Asians [170, 173, 174]. BKV subtype IV is particularly prevalent in East Asia [175], but has also been described in European populations [173, 176–179]. BKV subtype IV subgroups (IVa1, IVa2, IVc1, IVc2, IVb1 and IVb2) are found almost exclusively in Asia except IVc2 which occurs in Northeast Asia and Europe [180]. BKV subtyping has also provided insights into the mode of transmission. Second generation Japanese-Americans and Americans in California showed the European Ib2 lineage to predominate in both groups whereas Ic is most prevalent in Japan which suggests that transmission occurs outside the family [181]. There is no clear association with urinary excretion of a particular BKV subtype and human disease, and immunological status does not affect excretion of discrete BKV subtypes [13, 175]. 

### 4.2. BKV-Associated Clinical Disease

Following infection early in life, BKV remains latent in the tubular epithelium of the renal and urogenital tract [182]. Symptomatic reactivation of BKV in immunocompetent individuals is rare; however, the asymptomatic shedding of BKV in urine has been described in 7% of healthy adults without corresponding viraemia in paired plasma samples [183]. Three main clinical entities have been described associated with the BKV reactivation in the iatrogenically immunocompromised host: late-onset haemorrhagic cystitis, BKV nephropathy, and ureteral stenosis. 

Haemorrhagic cystitis (HC) is characterised by haemorrhage of the bladder mucosa with painful micturation which ranges from microscopic haematuria to clot retention and renal failure. HC-associated reactivation of BKV is a frequently encountered condition in immunocompromised haematopoietic stem cell transplant (HSCT) recipients leading to significant morbidity and occasional mortality [184]. HC is either an early-onset, preengraftment event arising from chemotherapeutic agents, particularly metabolites of cyclophosphamide and/or irradiation or a viral-associated postengraftment, late-onset event; the majority of which are due to reactivation of BKV but may also arise from cytomegalovirus and adenoviruses. Late-onset BKV-associated HC occurs in 6%–29% of HSCT recipients and normally two months after transplant [185]. Numerous studies have identified an association between reactivation of BKV, with both viruria and/or viraemia, and late-onset HC and an overall lowering of patient survival [186, 187]. Other authors have seen no significant difference between BKV viruria in HC and non-HC groups and only correlated-disease progression with high-level reactivation in HSCT groups [188]. A case-control study evaluating the association of BKV viraemia with HC in HSCT recipients showed that plasma viral load of >10^4^ copies/mL was detected in 63% of patients with HC and 57% of postengraftment BKV-HC cases compared with 5% of controls and importantly, BK viraemia occurred in 20 patients (67%) before clinical disease onset [189]. Saundh and colleagues have recently suggested that the monitoring of BKV viruria for early reactivation in the donor kidney may assist identifying patients at elevated risk of BKV-associated nephropathy (BKVN) [190]. 

BKVN develops in between 1%–10% of individuals who have undergone renal transplantation, generally within one year and up to 90% of these patients will lead to acute rejection [191]. Data from the United Network for Organ Sharing (UNOS; http://www.unos.org) show that graft loss attributable to BKVN was 7.5% (70/938) in 2009 and 5.7% (36/632) in 2010 [192].

Following the reactivation of latent BKV in the kidney, replication and lytic destruction of renal tubular epithelial cells occur resulting in tubular fluid accumulation in the interstitial compartment, characterised by an inflammatory interstitial nephropathy, associated with functional impairment due to tubular fibrosis and atrophy [193, 194]. No single risk factor has been definitively associated with BKVN in renal transplant recipients and the immunosuppressive regimen, and the intensity of immunosuppression appears to be the main factor resulting in BKV reactivation [13, 191]. With the triple immunosuppression and profound impairment of T-cell activation achieved by the increased usage of stronger calcineurin inhibitors such as tacrolimus, the use of antimetabolites like mycophenolate mofetil and anti-inflammatory corticosteroids has seen an increased incidence of BKVN [195]. A failure to mount or expand a cell-mediated immune response is further implicated in reactivation and replication of BKV, as interferon (IFN)-*γ* secreting BKV-specific T-cells were undetectable in renal transplant recipients who developed BKVN and correlated with higher levels of viraemia in BKV seropositive recipients [196, 197]. Strikingly, patients with BKVN treated by tapering of immunosuppression resulted in a reduction in plasma and urine viral loads, and the frequency of IFN-*γ*-secreting lymphocytes increased to the same level seen in healthy controls [196, 197]. *In vitro* investigations have also suggested that IFN-*γ* strongly inhibits replication/expression of BKV in primary human renal proximal tubule epithelial cells [198]. Taken together, the results suggest that cytokine and effector functions produced by cell-mediated immune responses are important in controlling viral reactivation and replication and clinical disease. 

Humoral immunity is thought to be less important as BKV seropositive patients prior to transplantation are not protected from viral reactivation, replication, and BKVN [199]. Donor antibody levels are inversely proportional to viruria onset and directly proportional to viruria duration and peak urine viral load indicating donor origin for early BKV infection in renal transplant recipients [200]. Finally, viral-associated factors have been implicated in BKVN, and BKV NCCR and VP1 mutations have been described; however, it is unclear whether this may simply arise from a preexisting lack of immune control of viral replication which would naturally lead to higher viral sequence diversity [201, 202]. Other risk factors identified for the development of BKVN include mismatched *HLA alleles*, advanced age, male gender, white ethnicity, diabetes, recipient seronegativity and lack of *HLA-C7* loci may also be associated with failure to control BKV replication [169, 193]. Interestingly, black renal transplant recipients had a lower risk of posttransplant BKV infection compared with white renal transplant recipients, independent of other confounding risk factors, suggesting that host factors that exist regulate viral latency and reactivation [203]. Genome wide association studies, such as have been conducted for hepatitis C virus to investigate ethnic differences in treatment responses, could conceivably be undertaken to potentially identify host genetic variation associated with poor prognosis [204]. 

Ureteral stenosis, necessitating percutaneous nephrostomy, has been associated with BKV viraemia when compared to aviraemic renal transplant recipients within one year of engraftment [205]. BKV-associated reversible upper urinary tract obstruction secondary to HC leading to ureteral stenosis has also been reported, though less frequently, in HSCT recipients [206]. 

Definitive diagnosis of BKVN requires a biopsy to be taken for histopathology to determine the severity of scarring, atrophy, interstitial fibrosis, and inflammation. However, as disease progresses following asymptomatic reactivation of latent virus in the kidney, monitoring for viruria and viraemia is undertaken by real-time PCR approaches—typically targeting the conserved T antigen gene—so a reduction in immunosuppression can be instigated early before extensive organ damage or allograft rejection can occur [207]. BKV-specific real-time PCR in plasma or sera are generally favoured over detection in urine as asymptomatic viruria is common and sustained viraemia is a better predictor for the development of BKVN [208]. Alternative approaches such as urinary cytology to detect renal tubular epithelial cells with intranuclear basophilic inclusion bodies on Papanicolaou staining (decoy cells) have low-positive predictive value in diagnosing BKVN compared to PCR-based approaches [208]. A cutoff of 10^4^ viral copies per mL of serum or plasma is commonly employed, and this approach of viral monitoring and tapering of immunosuppression to prevent development of nephropathy and graft dysfunction has been previously shown to be effective in cost-benefit analysis [209]. 

A potentially major breakthrough in the prevention and treatment of BKVN was suggested by a recent longitudinal serological study of kidney transplant recipients which demonstrated that BKV subtype I and subtype IV are serologically distinct using sensitive new methodologies [210]. In particular, the authors relied on BKV reporter vectors (pseudovirions) to evaluate serotype-specific neutralising antibodies rather than more traditional recombinant virus-like particles (VLP) ELISAs which crucially detect both neutralising and nonneutralising antibodies in the latter case. Using these antibody-mediated neutralisation assays, 5% and 49% of kidney transplant recipients were BKV subtypes I and IV naïve respectively pre-transplant, and 100% of BK subtype I and 43% of BK subtype IV seronegative patients pretransplant seroconverted in a type-specific manner. A model is presented where BKVN can arise from a *de novo* infection arising from a BKV subtype IV-infected kidney leading to replication in immunococompromised patients without prior exposure to this rarer BK subtype. Interestingly, prior studies have reported higher seroprevalence of BKV subtype IV in patients with interstitial nephritis [211]. Pastrana and colleagues argue persuasively that induction of a neutralising antibody response to BK subtype IV, or all subtypes, by vaccination of kidney transplant patients immunological naïve for certain subtypes prior to transplantation may prevent replication and BKVN associated with virus present in the transplanted organ [210]. 

## 5. Merkel Cell Polyomavirus

### 5.1. Epidemiology of MCV

In contrast to the lack of evidence for a strong and unambiguous association of other PyVs with human cancers, particularly, JCV and BKV, reviewed in [212], the Merkel cell polyomavirus (MCV) since discovery in 2008 has been strongly implicated in cellular transformation in an highly aggressive primary cutaneous neuroendocrine skin neoplasm (associated with a poor prognosis) termed Merkel cell carcinoma (MCC) [213, 214]. MCV shares a similar epidemiological profile to other human PyVs with serosurveys indicating that the exposure and infection occur early in childhood or asymptomatically later in life and that adult MCV seroprevalence is 60%–80% [215–217]. The precise mode of MCV transmission is unclear but as MC polyomaviral DNA (and HPyV6 and HPyV7) is found predominantly on human skin and shed in encapsidated virions, acquisition is most likely by respiratory or cutaneous routes [5]. 

### 5.2. MCC and Immunity

Heath and colleagues defined the most prominent clinical features of MCC in the acronym: AEIOU (asymptomatic/lack of tenderness, expanding rapidly, immune suppression, older than 50 years, and ultraviolet-exposed site on a person with fair skin), where 89% of primary MCCs had ≥3 of these findings [218]. Prior to the discovery of MCV, a defect in cellular immunity, indicative of an infectious disease aetiology for MCC, was suggested by a strikingly higher incidence (>13-fold increased risk) in HIV-infected individuals with clinical AIDS compared to the general population [219]. Notably, chronic lymphocytic leukaemia (CLL) was also found to be >30-fold overrepresented in MCC patients [218]. 

In addition, in association with iatrogenic immunosuppression, transplantation and MCC cases were reported following liver, heart, bone marrow and particularly renal allografts [220]. Discontinuation of cyclosporine and azathioprine immunosuppressive therapy and temporary regression of MCC metastases has also been reported [221]. Cases of MCC have also been seen in patients with a diverse array of autoimmune disorders, including systemic lupus erythematosus, chronic sarcoidosis, myasthenia gravis and Behçet's disease, correlating with the increased usage of potent immunosuppressive agents in the treatment of these conditions, such as fludarabine and rituximab which induce profound lymphopenia [222–225]. Age-specific incidence data for primary MCC also indicate that this is a disease of the elderly (90% of patients being older than 50 years) which correlates with age-related waning immune surveillance and impaired immunity [218]. Interestingly, there is a male predominance of MCC with a ratio of 1.4 : 1 (58.5% male and 41.5% female), and, increasingly, gender-based differences in inflammatory responses to pathogens are being recognized [218, 226]. Titres of anti-MCV antibodies are elevated in MCC patients suggesting that a defect in immune surveillance leads to viral replication and viraemia before tumorigenensis [227]. Adoptive immunotherapies may therefore offer promise for the treatment of MCV-MCC in elderly patients and other groups with impaired immunity as spontaneous remission of MCC has been reported which is thought to occur by T-cell-mediated immune response and tumour cell apoptosis [228].

Two mutational events following a loss of immune surveillance appear critical to cancer development in MCC patients; firstly, MCV is clonally integrated in an apparently unbiased location in the tumour genomes, and, secondly, the TAg helicase domain associated with NCCR binding and thus viral (lytic) replication is abolished; however, all mutations downstream of the LXCXE Rb tumour suppressor-binding motif are retained [4, 229]. The current model for MCC development is that some form of immune compromise (either age-related, iatrogenic, inherited; or infectious disease-related immunodeficiency) leads to a failure of cell-mediated immune surveillance of MCV, and virus integration into the host genome with abrogation of replicative capability through TAg mutation leading to clonal expansion. The discovery of MCV has led to better diagnostics for MCC but also critically the identification of potential treatments and the more rational design of therapeutics, for example, the identification of small molecule inhibitors of the survivin oncoprotein which was found upregulated following MCV binding of the tumour suppressor Rb [230]. 

## 6. KI Polyomavirus

KI polyomavirus (KIPyV, named for the Karolinska Institute in which it was first identified) was discovered as part of a systemic “molecular screening” search for unknown viruses in clinical respiratory tract samples in 2007 [2]. Using their own previously described methodology [231], the authors screened cell-free supernatants of 20 randomly selected nasopharyngeal aspirates that were submitted to the Karolinska University Laboratory for the diagnosis of respiratory tract infections. Of sequence reads from 374 clones, 75 were categorised as viral sequences: of these, 69 matched human rhinovirus or enterovirus species, 5 closely matched respiratory syncytial virus, and one showed weak amino acid similarity to the VP1 protein of the simian PyV SV40. The complete consensus viral genome sequence of this clone was determined from the original patient sample, identified as a polyomavirus and demonstrated on phylogenetic analysis to be clearly separate from all other known polyomaviruses [2]. Molecular prevalence studies performed on several sample sets detected KIPyV in 6/637 (1%) nasopharyngeal aspirates and 1/192 (0.5%) faeces samples but in none of 150 urine, 192 whole blood, 96 leucocyte, or 33 serum samples. Of interest, five of six KIPyV positive samples had another respiratory virus detected by standard diagnostic techniques (three RSV, influenza, and human metapneumovirus), suggesting that KIPyV may not have been responsible for the symptoms prompting nasopharyngeal sampling. 

Since its discovery, KIPyV DNA has been detected in respiratory specimens worldwide [232–237], suggesting widespread infection in humans. Indeed, Kean and colleagues have reported KIPyV seroprevalence rates of 55% in a population of healthy adult blood donors and paediatric blood samples. Of note, the seroprevalence in children 1–5 years of age (children <1 year were excluded to avoid the confounding effect of maternal antibody) was 44.6%, rising to 60.9% in 10–15 year olds, indicating that the primary exposure to the virus occurs in childhood [12]. In spite of KIPyVs widespread distribution, its pathogenicity or capacity to cause respiratory disease remains unconfirmed, as molecular prevalence studies in which control groups were included detected viral sequences at similar or higher frequencies in asymptomatic patients [236, 238]. In addition, the link between KIPyV and disease is complicated by the high rate of coinfection with other viruses [2, 232–234]. Nevertheless, the virus has been the sole pathogen identified in some cases [239]. KIPyV has been detected in blood from 4/130 (3.1%) healthy blood donors and in 2/62 (3.2%) HIV-infected individuals [240]. KIPyV has also been detected in the stool of patients with haematological disorders; however, the presence of other viruses capable of causing gastrointestinal disease makes causality difficult to establish. Nevertheless, an association between the presence of KIPyV and diarrhoea, compared to KIPyV-negative patients, has been reported [241]. The reported detection of KIPyV in the central nervous system [242] has not been confirmed, but the reason for the conflicting results is not known [243]. In order to determine if—like other HPyVs—KIPyV was more prevalent in immunocompromised patients, Kuypers and colleagues tested 2732 nasal washes during the first year after allogeneic HSCT from 222 patients [244]. After one year, the cumulative incidence estimate for KIPyV was 26%. Age <20 years and detection of a respiratory virus in the previous two weeks predicted KIPyV detection. Sputum production and wheezing were associated with detection of KIPyV in the past week. However, there was no association with acute graft versus host disease, CMV reactivation, neutropenia, lymphopenia, hospitalization, or death. The authors did not find a clear role for KIPyV (and WUPyV) as respiratory pathogens, and concluded that routine testing for these viruses in immunocompromised patients could not be recommended at this time [244]. 

## 7. WU Polyomavirus

WU polyomavirus (WUPyV, named for Washington University) was discovered in 2007 in a nasopharyngeal aspirate from a three-year-old child with pneumonia [3]. When standard respiratory virus PCR assays yielded no pathogen, total nucleic acid was randomly amplified, cloned, and shotgun sequenced. Of 384 reads obtained, six were classified as viral sequences that yielded three unique regions, each of which possessed limited homology to known PyV proteins. Subsequent analysis of the sequence data following the discovery of KIPyV revealed amino acid identities of 65% to 69% between WUPyV and KIPyV. Furthermore, an additional three of eight sequences that were previously unclassified demonstrated 58%–84% amino acid identity to KIPyV VP1 and VP2 proteins [3]. Molecular testing detected the novel virus in 37/1245 (3%) respiratory samples in 5/410 (1%) of upper respiratory specimens and in 1/480 (0.2%) bronchoalveolar lavage samples. Thirty-three of 37 positive specimens were from children under three years of age, and WUPyV was the sole virus detected in 12 patients with clinical evidence of respiratory tract infection. As with KIPyV, co-infections were common, with 25 of 37 samples yielding additional respiratory viruses, predominantly rhinovirus and human bocavirus. WUPyV DNA was not detected in any of 727 urine samples screened by PCR, the majority of which were obtained from renal transplant patients.

Since its discovery, WUPyV has also been detected in clinical samples worldwide [232, 234, 235, 237, 245–249]. Kean and colleagues reported WUPyV seroprevalence of 69% overall, from a baseline of 44.6% in 1–5 years olds, rising to 59.9% in 10–15 years olds and 70.9% in adults aged 50 years and older [12]. Again, this indicates the majority of human infection occurs in childhood. As for KIPyV, the pathogenicity of WUPyV given its detection in asymptomatic individuals remains to be resolved [243]. WUPyV has also been detected in blood [240], stool [241], and in unconfirmed reports in the CNS [242, 250], although, in contrast to KIPyV, the prevalence of WUPyV was higher in HIV-infected individuals (4.6%) than blood donors (0.8%) [240]. In allogeneic HSCT recipients, the cumulative annual incidence for WUPyV was 8%; as with KIPyV, age <20 years was predictive of detection [244]. Sputum production and wheezing were also associated with WUPyV detection in the preceding month. However, there was no association with acute graft versus host disease, CMV reactivation, neutropenia, lymphopenia, hospitalisation, or death. Routine testing of respiratory samples from immunocompromised individuals, as for KIPyV, is not recommended based on the currently available data [244].

## 8. Human Polyomavirus 6

Human polyomavirus 6 (HPyV6) was recovered in 2010 from the skin of healthy volunteers in a study designed to retrieve full-length wild-type MCV DNA from skin [5]. In addition to MCV, however, sequencing of the cloned rolling circle amplification (RCP) products also revealed the existence of two previously unknown PyVs, termed as HPyV6 and 7 (discussed below). Complete HPyV6 genomes were cloned from 5/35 individuals, with repeat sampling suggesting a chronic viral infection. Serology studies performed by Schowalter and colleagues on 95 samples from blood donors yielded an HPyV6 seroprevalence rate of 69%. These findings have been confirmed by others, with Nicol and colleagues reporting seroprevalence rates of 37.5% of 1–4 years olds, increasing to 61.8% in 15–19 years olds and 98.2% in those aged 80 years and older [215]. The increasing incidence with age suggests HPyV6 infection occurs throughout life. At present, there is no known disease association for HPyV6. However, viral DNA has been detected in faeces and nasopharyngeal swabs in transplant recipients [251]. The authors of the latter study note, however, that they cannot exclude contamination of these samples with virus shed from the skin.

## 9. Human Polyomavirus 7

Human polyomavirus 7 (HPyV7) was first identified on the skin of healthy volunteers enrolled in an MCV study as described above [5]. Complete HPyV7 genomes were cloned from 4/35 individuals, with repeat sampling suggesting chronic infection. The HPyV7 genome was 68% identical to HPyV6 at the nucleotide level. HPyV7 seroprevalence rates are lower than those of HPyV6. Schowalter's group reported HPyV7 seroprevalence rates of 35% in 95 adult blood donors, a finding confirmed by Nicol and colleagues, who reported rates of 10.4% in 1–4 years olds, increasing to 36% in 15–19 years olds, reaching 85.7% in individuals aged 80 years or more [215]. Again, the continued increase with age is indicative of infection occurring throughout life. HPyV7 has been detected in urine and nasopharyngeal swab samples from a liver transplant recipient [251]; however, there is no known disease association for HPyV7 at this time.

## 10. Trichodysplasia Spinulosa-Associated Polyomavirus

 Trichodysplasia spinulosa (TS) is an extremely rare (<1/1000000 prevalence) folliculocentric skin disease that has been described in iatrogenically immunocompromised hosts, particularly solid organ transplant recipients, and also in individuals with nontransplant-associated haematological malignancies, particularly acute lymphocytic leukaemia, receiving chemotherapy [6, 252–262]. TS is characterised by the development of predominantly facial, 1–3 mm follicular papules and keratotic protrusions (spicules or spines) often with accompanying alopecia of the eyelashes and brows [252, 254–256, 258–262]. An infectious aetiology for TS had been suspected since the description of 40–45 nm viral particles with transmission electron microscopy by Haycox (1999) and coworkers within the nuclei of the abnormally matured follicular inner root sheath cells overproducing trichohyalin [254]. 

The TS-associated polyomavirus (TSV), the eighth described human PyV, was identified by rolling circle amplification using the bacteriophage *φ*29 DNA polymerase and template derived from the plucked spicules of a TS patient [6]. The TS patient had received a standard immunosuppressive regimen (tacrolimus, mycopheolate mofetil and methylprednisolone) for cardiac transplant 18 months prior to presentation to dermatologists. Interestingly, the patient was treated with rituximab one year posttransplant, with concomitant tapering of immunosuppressive treatment, following the development of an EBV-positive B cell lymphoma [6]. The 5232-bp circular dsDNA TSV genome formed a monophyletic clade with Bornean orangutan PyV and was more closely related genetically to MCV than to BKV or JCV [6]. The 100% TSV positivity and active infection characterised by high viral titres (>10^6^ copies/cell) in skin lesions is strongly indicative of an aetiological relationship in disease pathogenesis [6, 263]. Age-specific seroprevalence studies in the human population have demonstrated that TSV is widespread in all age groups (41%–70% by age 10 and 70%–80% among adults) suggestive of primary exposure and the establishment of latency in early childhood with acquisition in adulthood a relatively rare event [215, 264, 265]. Furthermore, sensitive TSV-specific molecular assays failed to detect any active TSV infections in sera from a large elderly hospitalised population [266], and an age-specific decrease in anti-TSV antibody titres has also been observed [215]. Taken together, these findings suggest that TSV, like BKV, establishes a sub-clinical persistent infection early in childhood, that TSV does not replicate in adulthood in immunocompetent individuals and that progression from a latent to lytic cycle accompanies immunocompromise leading to active replication and associated disease. 

## 11. Human Polyomavirus 9 

Human polyomavirus 9 (HPyV9) was first discovered in the serum of a kidney transplant patient in 2011 [7]. Leendertz and colleagues screened 597 clinical samples collected from immunocompromised (renal transplant, HIV-infected, and PML) individuals, having previously identified more than 20 novel PyVs in non-human primates [267]. Phylogenetic analysis indicated that the HPyV9 genome was more similar to the genome of the African Green Monkey-derived lymphotropic polyomavirus (LPV) than to those of other PyVs. Interestingly, prior seroepidemiological studies had demonstrated that ≤30% of human sera had strong reactions to antigens derived from LPV [267, 268]. It appears that these findings can now be explained by cross-reactivity with HPyV9 [269, 270]. Nicol and colleagues reported HPyV9 seroprevalence rates of ~10% in clinical samples from children aged 1–7, rising to ~33% in healthy adult blood donors, the increasing prevalence with age suggesting that HPyV infection occurs throughout life. The same group later confirmed these findings in a larger patient cohort, with HPyV9 seroprevalence reaching 34% in 15–19 years olds and continuing to rise to 70% in subjects aged 80 years and older. Trusch and colleagues reported comparable seroprevalence rates in healthy children (13% in 2–5 years olds) and adolescents/young adults (35% in 11–20 years olds). Conversely, however, they also reported that HPyV9 seroprevalence peaked at 53% in 21–30 year olds, declining subsequently to 35% in subjects aged 60 years and older [270]. They also found a higher HPyV9 seroprevalence in renal and HSCT recipients, when compared with healthy controls. In contrast, liver transplant recipients and patients with neurological dysfunction demonstrated no such difference. HPyV9 DNA has also been detected in the urine of a child one week following liver transplant [251], and in respiratory samples from pregnant and nonpregnant females [271]. In contrast, HPyV9 DNA was not discovered in Japanese patients with CLL [272]. However, at present, there is no known disease association for HPyV9. Given that only five of the original 597 samples reported by Scuda and colleagues yielded positive HPyV9 DNA results by PCR (on repeat testing), and assuming an overall seroprevalence of 30%, it is probable that HPyV9—like other HPyVs—only causes disease in a small percentage of those infected (if at all).

## 12. MW Polyomavirus/Human Polyomavirus 10/MX Polyomavirus

Malawi polyomavirus (MWPyV) was identified by shotgun sequencing of DNA from virus-like particles isolated from a faeces sample collected from a healthy child from Malawi [9]. Siebrasse and colleagues subjected the purified DNA to 454 sequencing and identified six reads that aligned to tAg and VP1 proteins of known polyomaviruses. Phylogenetic analysis of the completed viral genome identified a novel polyomavirus that is highly divergent from other members of the *Polyomaviridae* family. Indeed, the different VP1 and VP2 tree topologies generated for MWPyV suggest it may be derived from an ancestral recombination event [9]. Molecular prevalence studies detected MWPyV in 12/514 (2.3%) stool samples from children presenting with diarrhoea. Interestingly, three of the positive samples were from a 5-year-old lung transplant recipient, taken over a period of four months, raising the possibility either of chronic infection or prolonged asymptomatic shedding: eight of the other nine patient samples were negative for all organisms tested, except MWPyV.

At the time the paper of Siebrasse's group was in press, another group was in the process of reporting a similar finding [8]. Buck and colleagues set out to identify unknown viruses in skin specimens taken from a patient with WHIM (warts, hypogammaglobulinemia, infection, and myelokathexis) syndrome, which is marked by an individual's relative inability to control human papillomavirus (HPV) infections [8]. Following rolling circle amplification, several cloned restriction fragments were homologous to various human and animal PyVs. Sequencing of the entire genome revealed what appeared to be a previously unknown human PyV, which the authors proposed to name HPyV10. However, subsequent comparative analysis of the nucleotide sequences of the MWPyV isolates demonstrated that they were from 95% to 99% identical to that of HPyV10, and as such constitutes two strains of the same viral species rather than two separate novel species.

 Following these two reports, a third paper subsequently appeared reporting the discovery of a novel PyV in acute diarrhoeal samples from children [273]. Yu and colleagues employed an unbiased deep sequencing approach to identify a novel highly divergent HPyV in stool samples from children. The initial sequence was discovered in a sample from a two-year-old child with diarrhoea from Mexico, hence the proposed name MXPyV. The virus differed substantially from the other nine known PyVs, with amino acid sequence identities ranging from 13%–44%. Subsequent molecular prevalence studies performed by the group detected MXPyV in 12/96 (12.5%) stool samples from children in Mexico, in 18/546 (3.3%) stool samples in California, and in 4/96 (4.2%) in Chile [273]. However, no association between MXPyV and the presence of diarrhoeal symptoms was noted. MXPyV was also detected in 1/136 (0.74%) respiratory samples from hospitalised children with pneumonia in Mexico. MXPyV was not detected in any of 480 plasma and urine samples from renal (*n* = 283) or solid organ and bone marrow transplant (*n* = 193) recipients. When the complete sequence of MXPyV was compared with the novel MWPyV/HPyV10 above, it transpired that it was almost identical, sharing 99.8% or 99.7% identity, respectively. As such, the three viruses are different variants of the same species, and likely represent the first members of a new subclade of PyVs. Nevertheless, the seroprevalence of this virus in different human populations remains unknown, as does its capacity to cause disease. 

## 13. Polyomavirus-Encoded MicroRNAs: Immune Evasion, Establishment of Persistent Infections, and Potential Therapeutic Applications

MicroRNAs (miRNAs) are small (~22 nucleotide), non-coding, posttranscriptional regulators of gene expression, initially identified in model organisms, and subsequently described in metazoans and viruses [274, 275]. Seminal work from the Sullivan Laboratory identified the existence of an SV40 virally-encoded miRNA in an antisense orientation to the TAg early gene target leading to an autoregulatory loop and downregulation of TAg mRNA and evasion from CD8+ cytotoxic T lymphocyte (CTL) immune surveillance [276]. These monkey SV40-encoded miRNAs were subsequently shown to be evolutionarily conserved in both sequence and function in the human pathogens BKV and JCV [277]. Intriguingly, these miRNAs are expressed in JCV PML brain tissue which suggests that the viral miRNAs could be therapeutic targets using appropriately delivered synthetic oligonucleotides, that is, anti-miRNAs, termed antagomirs [277, 278]. 

This was extended by elegant work which demonstrated that a viral miRNA identical in sequence between BKV and JCV was shown to target the stress-induced ligand ULBP3—a protein recognized by the natural killer (NK) cell receptor NKG2D [279]. BKV and JCV miRNA-mediated downregulation of ULBP3 decreased NKG2D-mediated killing of virus-infected cells by NK cells. Conversely, inhibition of the viral miRNA during infection leads to enhanced NK cell killing of infected cells implicating these virally-encoded miRNAs in the establishment and maintenance of lifelong persistence by compromising the host immune system [279]. MCV has also been shown to encode a miRNA from the late strand in antisense orientation to early viral transcripts in 50% of Merkel cell tumours and predicted to target host mRNAs leading to immune evasion [280, 281]. Moreover, in MCC, MCV-encoded microRNAs were identified that potentially regulate T- and B-cell receptor signalling hampering viral (tumour) immune recognition [280]. 

It is unclear whether detection of virally encoded miRNAs has diagnostic or prognostic clinical utility—in an analogous manner to the detection of BKV DNA in serum and plasma and VP1 mRNA in urine samples or JCV DNA in CSF—and longitudinal studies of immunocompromised renal-transplant recipients and patients receiving immunomodulatory agents are of interest to see whether PyV miRNA presence or absence in different compartments precede development of PyV-associated disease.

## 14. Antiviral Treatment

At present, there is no antiviral therapy specifically licensed for the treatment of either JCV or BKV infections. Despite anecdotal reports of response to various treatments in the literature, all controlled studies have failed to show any efficacy for the drugs tested against PML [23]. This includes cidofovir (CDV), cytosine arabinoside (Ara-C), and mefloquine. However, as DNA viruses, many of the available DNA polymerase inhibitors exhibit a level of *in vitro* activity against PyVs and have been used in the clinical setting [147]. To date, with the exception of TSV, there is little information of the benefit of antiviral therapy for the majority of the novel HPyVs, and the available evidence base is outlined in Table 2. 

Cidofovir (CDV) is an acyclic nucleotide phosphonate analogue of deoxycytosine monophosphate licensed for the treatment of CMV retinitis, which has shown *in vitro* activity for non-human PyVs and BKV in cell culture, although a study employing JCV and a human neuroglial cell line showed no significant effect on replication [292]. Subsequent case reports in the literature suggested a possible clinical benefit from CDV in the treatment of PML [293, 295, 319]. However, larger case series and an analysis of six international cohorts of HIV-infected individuals affected by PML showed no significant impact of CDV on overall survival [291, 296, 297, 320]. It should be highlighted that all clinical studies to date have been retrospective or observational [147]. As such, to reach a definitive conclusion on the efficacy of CDV for PML, a randomised control trial (RCT) is needed. In contrast, CDV has improved clinical outcomes and decreased viruria and viraemia in BMT patients with BKV-associated HC [294]. In addition, low-dose CDV has been associated with enhanced graft survival in renal transplant patients with BKV interstitial nephritis [282]. However, the drug has limited treatment potential in renal transplant patients due to its toxicity and its limited oral bioavailability [283]. In this respect, of interest is the recent development of CMX001, an orally administered, bioavailable hexadecyloxypropyl lipid conjugate of CDV with reduced nephrotoxicity. CMX001 was found to reduce JCV replication by as much as 60% in human cell lines [284, 285]. It has also been successfully used in a patient with PML and idiopathic CD4+ lymphocytopenia [286]. While both *in vitro* studies reported decreasing cell viability with increasing concentrations of CMX001, suggesting toxicity remains an issue for this drug, Chimerix has recently reported that the drug is well tolerated in healthy volunteers at doses up to 2 mg/kg [287]. CMX001 is also able to inhibit BKV replication in human renal proximal tubule epithelial cells more rapidly and with a longer-lasting effect than CDV [288]. A double-blind randomised placebo-controlled trial of CMX001 in posttransplant subjects with BKV viruria has recently been completed and results are awaited (ClinicalTrials.gov-NCT00793598). Finally, topical CDV gel has been used to successfully treat TSV infection in a 15-year-old male heart transplant recipient and a 57-year-old woman with CLL [6, 289].

Ara-C is a nucleoside analogue that was effective in decreasing JCV replication in cultured human neuroglial cells [292]. In the clinical setting, reports in the literature have described positive [290, 298, 299, 321] and negative [300, 301, 322, 323] results. The only randomised clinical trial to date (ACTG 243) demonstrated that Ara-C administered either intravenously or intrathecally did not prolong survival for PML patients [302]. 

JCV enters host cells through binding of the virus to the primary receptor *α*2,6-linked sialic acid moieties and the secondary receptor serotonin receptor 2A (5HT_2A_R) [303]. Consequently, blocking access to this receptor—either through the use of antibodies or serotonin receptor antagonists—has been studied as a potential therapeutic approach for PML. To date, *in vitro* studies have demonstrated that ketanserin and ritanserin are effective at limiting JCV infection in human brain-derived cell culture [304]. In addition, newer antipsychotics such as risperidone and olanzapine have been reported to be up to ten times more potent *in vitro* at inhibiting JCV infection [305]. Furthermore, treatment of PML with mirtazapine alone and in combination has been associated with favourable outcomes [299, 306]. However, subsequent *in vitro* studies have shown that 5HT_2A_R is not essential for JCV infection of certain cells in the human brain [307, 308], and the only clinical report of a non-HIV PML case treated with chlorpromazine and CDV did not show any clinical benefit [309]. Consequently, the role of serotonin receptor antagonists in the treatment of PML remains to be determined.

Mefloquine is an antimalarial drug that has been shown to inhibit JCV replication *in vitro*. The viral target of the drug is unknown but may directly inhibit the PyV T antigen [310]. Initial case reports showed that mefloquine treatment of PML was successful in lowering the viral burden in the brain and improved clinical symptoms [311, 312]. However, a multicentre clinical trial of mefloquine in PML patients failed to demonstrate a reproducible reduction in JCV DNA in PML patients or reduced clinical progression of PML [23].

Leflunomide is a calcineurin inhibitor (immunosuppressant) licensed for the treatment of rheumatoid arthritis known to block dihydroorotate dehydrogenase, tyrosine kinase, and pyrimidine synthesis. In addition, it has been reported to have modest *in vitro* activity inhibiting BK viral replication [313, 314]. In clinical studies, leflunomide alone, in combination with CDV, decreased urine and plasma BK viral loads in renal transplant recipients, although drug monitoring is required to ensure adequate therapeutic levels [315]. However, not all studies have demonstrated a benefit [316, 317], and some argue that the addition of drug therapy in treatment of BKVN provides no benefit compared with the standard of care, that is, the reduction of immunosuppression alone [318].

The fluoroquinolones (FQ) are a class of antibiotics that have been used—in combination with immunosuppression reduction—in the treatment of BKVN and have been shown to have *in vitro* activity against BKV [324]. These antibiotics inhibit bacterial DNA replication by inhibiting type II topoisomerases and are thought to have activity against the viral helicase TAg [325]. In clinical studies, ciprofloxacin prophylaxis has been shown to reduce the rate of BK viraemia in renal transplant recipients [326, 327] and to reduce the incidence of severe HC in allogeneic HSCT [328]. However, the long-term effectiveness and optimal duration of FQ prophylaxis against BKV infection remain unknown [327]. 

Mammalian target of rapamycin (mTOR) inhibitors such as sirolimus reduce translation and cell cycle progression and reduce BKV replication *in vitro* [329]. An initial retrospective study, albeit with small numbers, suggested that renal allograft recipients on mTOR inhibitors cleared BK viruria and viraemia more quickly than those on other immunosuppressive agents [330]. However, other reports have not supported these findings [207, 331]. It may be possible that mTOR inhibitors are preferable to calcineurin inhibitors from the perspective of reducing the risk of BKV-associated disease, but at this point, there are no RCT data to suggest a benefit from mTOR inhibitors as antiviral treatments in this setting [13].

While there is no single reason for the lack of effective antiviral therapy for PyV infection, there is probably a number of challenges that explain the existing dearth. First, should the drug target the virus or aim to stimulate the host immune system? Second, if the virus is the target, which stage of the life cycle should be chosen: the primary infection, latency-site cell entry, or virus replication? Prevention of primary infection would only be achievable through large-scale population immunization, an approach that is not likely to be cost effective; while the inhibition of latency-site cell entry may be feasible (some virus receptors have been identified as mentioned above), the impact of such agents on preexisting latent virus is likely to be negligible. Thus, successful therapies will have to retard intracellular virus replication, requiring sufficient doses of active drug to be able to cross the BBB without causing significant toxicity, a significant problem for existing agents. Third, would a single specific antiviral agent be effective against both “prototype” and “archetype” JC virus, for example, or could such an agent drive the emergence of resistance? Fourth, given these unknowns, not to mention the relatively small number of suitable patients requiring antiviral treatment (and thus available for RCTs), is it reasonable to expect a single pharmaceutical company to take on the economic risk of developing such an agent, or will government input be required? Furthermore, if government input is required, would that constitute an appropriate allocation of limited resources in the current straitened times? If HIV-infected patients still constitute the population at greatest risk of PML for example, and the majority can be successfully treated with combination ART; perhaps, the more prudent approach to the management of PyV infected individuals is a risk reduction strategy, as evidenced by the prescreening of MS patients for JC antibodies before commencing natalizumab therapy. In summary, there are no currently available RCT data to recommend the use of specific antiviral therapy for the treatment of PyV infection, although the commencement of antiretroviral therapy is recommended in HIV-associated PML. For non-HIV PML and BKV disease in transplant recipients, withdrawal from, or reduction in, immunosuppression—insofar as is possible—is the present mainstay of treatment.

## Figures and Tables

**Table 1:** The human polyomaviruses, associated disease and immunocompromised risk groups.

Human polyomavirus	Adult seroprevalence	Clinical disease	Patient's risk groups
JCV	50%–80%	Progressive multifocal leukoencephalopathy	HIV-infected, immunomodulatory therapies
BKV	≥90%	BKV nephropathy, haemorrhagic cystitis, ureteral stenosis	Solid organ and HSCT transplant recipients
MCPyV	60%–80%	Merkel cell carcinoma	>50 years of age, immune suppression
WUPyV	≥69%	No strong association	Not defined
KIPyV	≥55%	No strong association	Not defined
HPyV6	≥83%	No strong association	Not defined
HPyV7	≥64%	No strong association	Not defined
TSV	70%–80%	Trichodysplasia spinulosa	Transplant recipients, immune suppression
HPyV9	34%–70%	No strong association	Not defined
MWPyV	Not defined	No strong association	Not defined

**Table 2:** Antiviral therapies for human polyomavirus infection.

Antiviral agent	Mechanism of action	HPyV	*In vitro* activity	Clinical syndrome	Study design	Patient group	Clinical benefit	References
				—	—	—	—	[274]
Cytosine arabinoside (Ara-C)	Nucleoside analogue (DNA polymerase inhibitor)	JCV	Yes	PML	Case series/reports	HIV, non-HIV, dermatomyositis	Yes	[282–285]
				PML	Case series/reports	HIV, non-HIV, dermatomyositis	No	[282, 286–289]
				PML	RCT	HIV	No	[290]

				—	—	—	—	[274]
				PML	Pilot study	HIV	No	[281]
		JCV	No	PML	Multicohort analysis	HIV	No	[279]
				PML	Multicentre Retrospective analysis	HIV	No	[278]
Cidofovir (CDV)	Nucleotide phosphonate analogue (DNA polymerase inhibitor)			PML	Case series	HIV	No	[280]
				PML	Case reports	HIV, the Heerfordt syndrome, SLE	Yes	[275–277]
				—	—	—	—	[291]
		BKV	Yes	HC	Case series	HSCT	Yes	[292]
				BKVN	Nonrandomised Controlled study (low-dose CDV)	Renal T'plant	Yes	[293]
		TSV	N/A	TS	Case report	Heart T'plant, CLL	Yes	[6, 294]

		JCV	Yes	—	—	—	—	[295, 296]
				PML	Case report	Idiopathic CD4+ lymphocytopaenia	Yes	[297]
CMX001	Lipid conjugate of Cidofovir							
		BKV	Yes	—	—	—	—	[291]
				BK Viruria	RCT	Renal T'plant	Pending	ClinicalTrials.gov-NCT00793598

				—	—	—	—	[298, 299]
				PML	Case report (mirtazapine)	Dermatomyositis	Yes	[285]
Serotonin receptor 2A (5HT_2A_R) antagonists	Inhibits JCV receptor binding and cell entry	JCV	Yes	PML	Case report (mirtazapine)	HIV	Yes	[300]
				PML	Case report (chlorpromazine)	CLL/HSCT	No	[301]

Mefloquine	Unknown: may directly inhibit large T antigen	JCV	Yes	—	—	—	—	[302]
				PML	Case report	Sarcoidosis, AML/UBCT	Yes	[303, 304]
				PML	RCT	HIV	No	[23]

Leflunomide	Inhibits pyrimidine synthesis; inhibits tyrosine kinase	BKV	Yes	—	—	—	—	[305, 306]
				BKVN	Case series	Renal T'plant	Yes	[307]
				BKVN	Case series	Renal T'plant	No	[308]
				BKVN	Case series	Renal T'plant	No	[309]

FK778	Derived from the active metabolite of Leflunomide			BKVN	RCT	Renal T'plant	No	[310]

Fluoroquinolones	Inhibit large T antigen helicase activity	BKV	Yes	—	—	—	—	[311, 312]
				BK viraemia	Case series	Renal T'plant	Yes	[313]
						Renal T'plant	Yes (3 mo.)	[314]
							No (12 mo.)	[314]
				HC	Case series	HSCT	Yes	[315]

Mammalian target of rapamycin (mTOR) inhibitors	Reduce translation and cell cycle progression	BKV	Yes (sirolimus)	—	—	—	—	[316]
				BKVN	Case series	Renal T'plant	Yes	[317]
				BKVN	Case report	Renal T'plant	No	[199]
				BK viraemia	Prospective Nonrandomised controlled study	Renal T'plant (Paediatric)	No	[318]

This table lists the available therapeutic options for human polyomavirus infections. KIPyV, WUPyV, HPyV6, 7, 9, and 10 have not been included as there are presently no definitive disease associations for these viruses. Abbreviations are defined in the accompanying text.
